# Agronomic and Physiological Aspects of Programmed Cycle Pruning in *Coffea arabica*

**DOI:** 10.3390/plants15111597

**Published:** 2026-05-22

**Authors:** Diego Corona Baitelle, Sílvio de Jesus Freitas, Henrique Duarte Vieira, Abraão Carlos Verdin Filho, Sávio da Silva Berilli, Ismael Lourenço de Jesus Freitas, Weverton Pereira Rodrigues, Danilo Força Baroni, Silvério de Paiva Freitas, Guilherme Bessa Miranda, Stella Arndt, Orlando Carlos Huertas Tavares, Leandro Pin Dalvi, Paulo Cesar dos Santos

**Affiliations:** 1 Center for Agricultural Science and Technologies, Northern Fluminense State University Darcy Ribeiro (UENF), Campos dos Goytacazes 28013-602, RJ, Brazil; dg.corona@gmail.com (D.C.B.); silvio@uenf.br (S.d.J.F.); henrique@uenf.br (H.D.V.); verdin.abcfilho@gmail.com (A.C.V.F.); baronidf@gmail.com (D.F.B.); silverio@uenf.br (S.d.P.F.); 2 Sustainable Agriculture Laboratory, Federal Institute of Education, Science and Technology of Espírito Santo (IFES), Alegre Campus, Alegre 29500-000, ES, Brazil; savio.berilli@ifes.edu.br; 3 Instituto Capixaba de Pesquisa, Assistência Técnica e Extensão Rural (INCAPER), Vitória 29052-010, ES, Brazil; ismaelljf@yahoo.com.br (I.L.d.J.F.); gbm3009@gmail.com (G.B.M.); 4 Center of Agricultural Sciences, State University of the Tocantins Region of Maranhão (UEMASUL), Imperatriz 65900-001, MA, Brazil; weverton.rodrigues@uemasul.edu.br; 5 Department of Agronomy, Federal University of Espírito Santo, Alegre 29500-000, ES, Brazil; stella.arndt@edu.ufes.br (S.A.); leandro.dalvi@ufes.br (L.P.D.); 6 Department of Technical Education in Agriculture, Fluminense Federal Institute of Education, Science and Technology, Cambuci 28430-000, RJ, Brazil; ochtavares@gmail.com

**Keywords:** canopy stratification, plant architecture, stomatal conductance, leaf pigments, instantaneous leaf-level water use efficiency, crop productivity, tropical coffee systems

## Abstract

Programmed Cycle Pruning (PCP) in Arabica coffee can positively influence plant physiology by modifying plant architecture, promoting a more uniform distribution of branches and leaves, and altering microclimatic conditions within the canopy, particularly light incidence. These structural changes may contribute to improvements in plant performance and productivity. The objective of this study was to evaluate growth, yield, and physiological responses of Arabica coffee plants managed under PCP at different stem densities per hectare. The experiment was conducted in a randomized block design with four replications. Treatments were arranged in a 4 × 2 factorial scheme with an additional treatment representing the traditional pruning system. The factorial combination included four stem densities (4000, 8000, 12,000, and 16,000 stems ha^−1^) and two data collection positions on the plant (lower and upper canopy strata). The evaluated variables included canopy diameter, plagiotropic branch length, number of inflorescences per branch, net photosynthetic rate (Anet), stomatal conductance (gs), leaf transpiration (E), vapor pressure deficit between leaf and air (VPDleaf/air), SPAD index, anthocyanin and flavonoid contents, and grain yield. PCP promoted greater uniformity in leaf gas exchange within the canopy and prevented the occurrence of “girdling”, which under traditional pruning reduced Anet in the upper canopy. Net photosynthesis increased with stem density under PCP. Although growth variables were similar between pruning systems, yield was higher under PCP, with a nonlinear response to stem density, indicating improved canopy gas-exchange uniformity and productivity in Arabica coffee cultivation.

## 1. Introduction

Coffee is a crop of great socioeconomic importance for Brazil, and among the cultivated species, Arabica coffee (*Coffea arabica* L.) remains the most relevant in the productive and commercial chain, often accounting for the largest share of national production. In the 2025 harvest, according to the final estimate of the 4th Coffee Crop Survey (December 2025), Arabica coffee recorded a national average yield of 1.45 t ha^−1^, reflecting the effects of the negative biennial production cycle and adverse climatic conditions on its productive potential. Even so, Arabica coffee maintained a central role in Brazilian coffee farming, highlighting its significant economic and productive importance in the country in 2025 [1].

The low average yield of Arabica coffee stimulates the search for technologies and management strategies capable of increasing productivity. Among the available technologies, pruning has great potential to enhance Arabica coffee yield [2]. In addition to being widely accepted and accessible to growers, pruning helps maintain productive capacity, recover weakened plants, correct problems related to plant architecture, reduce disease severity, increase crop longevity, and contributes to reducing production biennially [3,4,5].

Traditional pruning systems in Arabica coffee, such as topping, skeleton pruning, and stumping, are often associated with drastic canopy removal, temporary loss of productivity, and, in some cases, a “zero yield year”. In addition, these practices may reduce root system activity and delay plant recovery, limiting their effectiveness in sustaining long-term productivity. This limitation is closely related to the biennial production pattern in Arabica coffee, characterized by alternating years of high and low yield due to the depletion of plant reserves during high-yield seasons. Among the pruning systems used in Arabica coffee, Programmed Cycle Pruning (PCP) has emerged as a promising alternative, being considered a viable technique from both productive and economic perspectives [2,6]. This system, also referred to in Brazilian literature as “poda programada de ciclo (PPC)”, is based on the cyclical renewal of orthotropic stems, in which a defined number of stems per plant is maintained according to plant spacing, resulting in controlled stem density per hectare. The system involves the periodic removal of older stems and highly productive plagiotropic branches, combined with the gradual replacement of stems over successive production cycles. This strategy was originally developed for *Coffea canephora* (Conilon coffee) and later adapted for Arabica coffee, aiming to improve plant architecture, longevity, and productivity.

PCP promotes the formation of multi-stem plants with a more open canopy architecture, allowing greater light penetration into the inner canopy and improving the distribution of branches and leaves. Increased light availability in the inner canopy can stimulate the emission of vigorous basal shoots, contributing to continuous canopy renewal. In addition to structural changes, this system may contribute to extending the productive lifespan of coffee plantations by promoting continuous structural renewal.

In PCP, older plant tissues are removed, which stimulates the formation of new branches and leaves. In addition, the elimination of plagiotropic branches may lead to increases in the net carbon assimilation rate of the remaining foliage [7], due to the enhanced light interception capacity of the plants, a key environmental factor in agricultural production [8,9]. Unlike traditional pruning systems, which may lead to a “zero yield year” due to drastic canopy removal, PCP enables more stable annual production by maintaining part of the productive structure during renewal cycles and may contribute to reducing yield biennially. This pruning system may promote a better distribution of photoassimilates within the plant, since it does not lead to the formation of the phenomenon known as “girdling,” which is commonly observed under traditional pruning systems. This condition is associated with a source–sink imbalance, in which the lower canopy acts as a dominant sink, which may restrict assimilate allocation to upper plant regions and impair overall canopy development [10].

Photosynthetic capacity is also influenced by the chlorophyll content of the leaves [8], which is directly related to nitrogen content. Therefore, the removal of branches in the lower third of the plant may alter the source–sink relationship, improve the distribution of photosynthates and nutrients, and increase productivity. Despite these potential physiological advantages, the application of this system in Arabica coffee remains poorly understood, particularly regarding its effects on plant physiology, growth, and yield under different management conditions. Therefore, there is still limited information available to support these mechanisms. The PCP system is expected to promote greater physiological uniformity within the canopy by improving light distribution and source–sink balance, resulting in higher net photosynthesis and increased grain yield compared with the traditional pruning system.

In this context, the objective of this study was to evaluate the growth, yield, and physiological aspects of Arabica coffee managed under the Programmed Cycle Pruning system at different stem densities per hectare, as well as under the traditional pruning system. We hypothesize that PCP modifies canopy architecture in *Coffea arabica* by improving light distribution and reducing source–sink imbalance within the plant, thereby promoting greater physiological uniformity between canopy strata. This structural reorganization is expected to mitigate the occurrence of girdling, enhance net carbon assimilation efficiency, and optimize the allocation of photoassimilates throughout the plant. Consequently, these integrated physiological and architectural adjustments should result in increased grain yield compared to the traditional pruning system, with stem density playing a key role in modulating the magnitude of these responses.

## 2. Results

### 2.1. Conventional Pruning vs. Programmed Cycle Pruning (PCP): Canopy Analyses

The PCP system (factorial) and the traditional pruning system (control) showed similar values for leaf gas exchange. Net photosynthetic rate (A_net_) averaged 5.5 µmol m^−2^ s^−1^ (±0.18), transpiration (E) 1.39 mmol m^−2^ s^−1^ (±0.065), and stomatal conductance (g_s_) 0.066 mol m^−2^ s^−1^ (±0.002). The SPAD index also remained similar between systems, with a mean value of 63.66 (±0.67). No differences were detected between pruning systems for any of the evaluated variables.

The responses of shoot growth were similar in both pruning systems. Canopy diameter, branch length, and number of inflorescences per branch did not differ between treatments. The factorial treatment presented mean values of 0.96 m (±0.27), 0.48 m (±0.14), and 8.94 (±0.66), whereas the control showed mean values of 1.00 m (±0.25), 0.50 m (±0.12), and 9.37 (±1.07) for canopy diameter, branch length, and number of inflorescences per branch, respectively.

Grain yield differed between pruning systems, with higher values observed under PCP compared with the control. Plants managed under PCP showed a yield of 2.25 t ha^−1^, whereas the control treatment, managed under the traditional pruning system, produced 1.59 t ha^−1^, representing an increase of 0.67 t ha^−1^.

**Figure 1:**
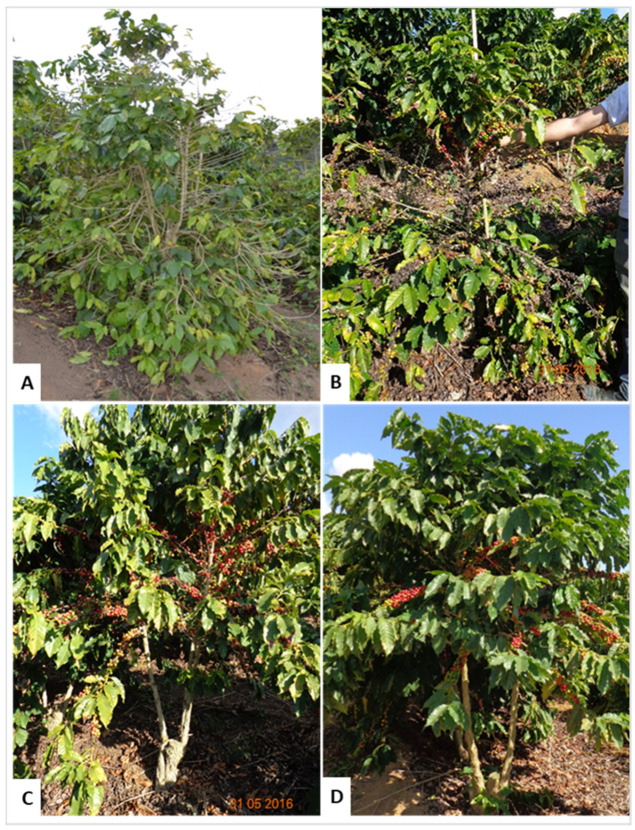
Presence of girdling in the control treatment (**A**,**B**), and absence of girdling in the other treatments for Arabica coffee. Programmed cycle pruning with three stems and annual (**C**) and biennial (**D**) removal of plagiotropic branches that had reached 70% or more of their production.

**Figure 2:**
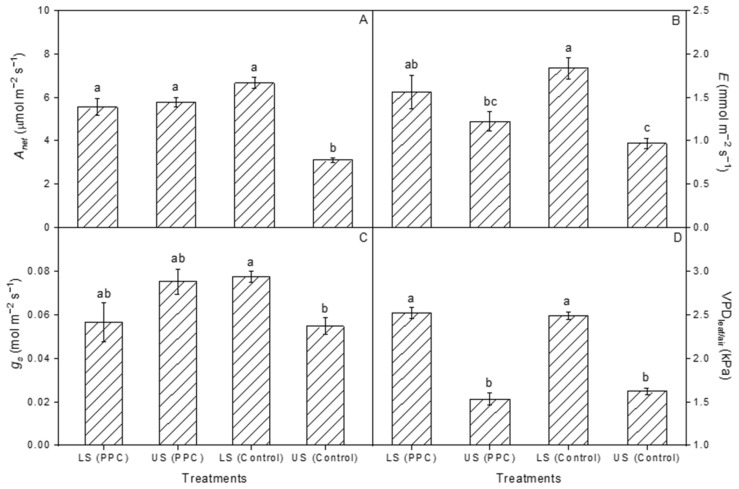
(**A**) Net photosynthetic rate (A_net_), (**B**) transpiration (E), (**C**) stomatal conductance (g_s_), and (**D**) vapor pressure deficit between leaf and air (VPD_leaf/air_) as a function of data collection position (LS = lower stratum and US = upper stratum) in Arabica coffee under the PCP system and the conventional pruning system (control). Mean values ± SE (n = 4) followed by the same letter do not differ significantly according to Tukey’s test (*p* ≤ 0.05).

Plants under PCP exhibited a more uniform distribution of branches within the canopy compared with those managed under the traditional pruning system (Figure 1). Based on this structural difference, the canopy was subdivided into lower and upper strata for data collection in order to evaluate differences in canopy stratification and their association with yield (Figure 2).

### 2.2. Traditional Pruning vs. PCP: Analyses of Plant Strata

A_net_ in the upper stratum (US) of the control treatment showed a reduction of approximately 53.45% and 43% compared with the lower stratum (LS) of the same plants and with the US and LS of the treatments under the PCP system, respectively (Figure 2A). On the other hand, under the PCP system there was no difference (*p* ≤ 0.05) in Anet between plant strata, with the US presenting 5.7 µmol m^−2^ s^−1^ and the LS 5.5 µmol m^−2^ s^−1^.

The low A_net_ observed in the US (control) was also accompanied by low E (0.96 mmol m^−2^ s^−1^) (Figure 2B) and low g_s_ (0.055 mol m^−2^ s^−1^) (Figure 2C). Conversely, in the LS (control), the high A_net_ was accompanied by higher E (1.83 mmol m^−2^ s^−1^) and g_s_ (0.078 mol m^−2^ s^−1^). This pattern was not observed in plants under the PCP system, as A_net_, E, and g_s_ were statistically equal (*p* ≤ 0.05) between plant strata. Greater uniformity in leaf gas exchange was observed under PCP compared with the traditional pruning system. However, US (PCP) showed lower E (1.22 mmol m^−2^ s^−1^) than LS (control) (1.83 mmol m^−2^ s^−1^), whereas A_net_ and g_s_ were statistically equal (*p* ≤ 0.05).

**Figure 3:**
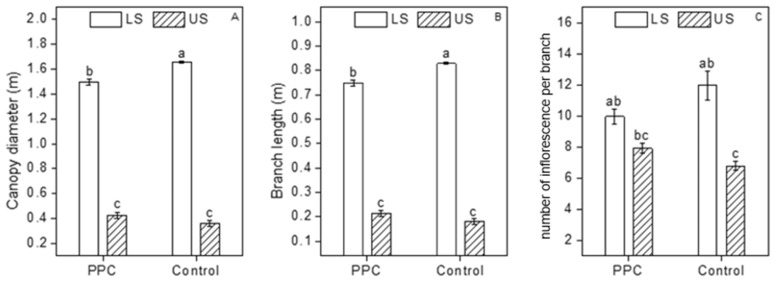
(**A**) Canopy diameter, (**B**) branch length, and (**C**) number of inflorescences per branch as a function of data collection position (LS = lower stratum and US = upper stratum) in Arabica coffee under the PCP system and the traditional pruning system (control). Mean values ± SE (n = 4) followed by the same letter do not differ significantly according to Tukey’s test (*p* ≤ 0.05).

Canopy diameter and branch length were significantly greater in the LS compared with the US, whereas the number of inflorescences per branch differed only in the control treatment (Figure 3). In the PCP system, the US showed canopy diameter and branch length values 3.4 times smaller than those of the LS, whereas in the control this difference was 4.5 times. This variation between strata was observed regardless of the pruning system.

The SPAD reading in the US (57.87) and LS (66.26) indicated a difference in chlorophyll synthesis between plant strata, with the US showing a lower index than the LS for both pruning systems (Table 1). There was no difference (*p* ≤ 0.05) in the LS between pruning systems, whereas in the US, control plants showed a lower index than those subjected to the PCP system.

**Figure 4:**
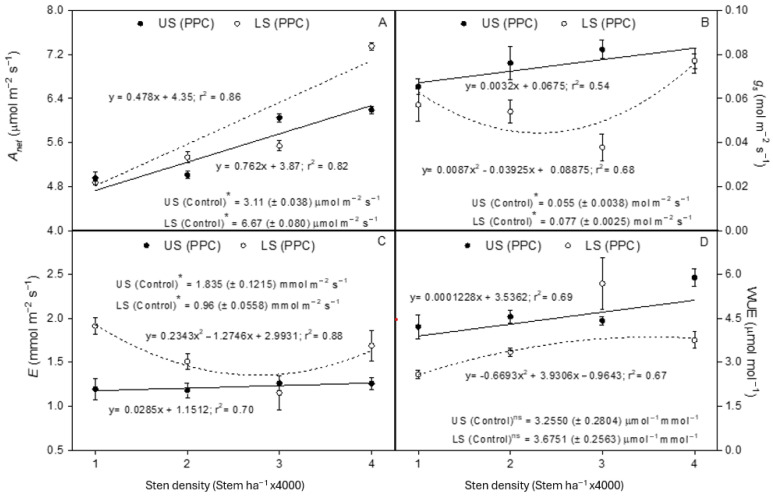
(**A**) Net photosynthetic rate (A_net_), (**B**) stomatal conductance (g_s_), (**C**) transpiration (E), and (**D**) instantaneous leaf-level water use efficiency (WUE) in Arabica coffee managed under the PCP system with different stem densities ha^−1^ and under the traditional pruning system (control). US = upper stratum and LS = lower stratum. (*) Significant (*p* ≤ 0.05) and (NS) not significant (*p* ≥ 0.05). Each value represents the mean ± SE (n = 4).

In the PCP system, there was a significant interaction (*p* ≤ 0.05) between stem density ha^−1^ and plant stratum for leaf gas exchange measurements and instantaneous leaf-level water use efficiency (WUE) (Figure 4). In both strata, A_net_ increased linearly with the increase in stem density ha^−1^, reaching 6.1 and 7.3 µmol m^−2^ s^−1^ in the US and LS, respectively, at 16,000 stems ha^−1^. This represented an increase of 25% and 50% compared with the lowest observed value, respectively (Figure 4A). Higher Anet values were observed in the LS compared with the US across stem densities.

In the US, there was a strong correlation among A_net_, g_s_, and E (Table 2), influenced by the increase in stem density ha^−1^ (Figure 4A–C). However, the same behavior was not observed in the LS, since the increase in A_net_ (resulting from the increase in stem density ha^−1^) was independent of variations in g_s_, which, in turn, showed no correlation with the increase in stem density ha^−1^.

Furthermore, in the US, the increase in stem density ha^−1^ promoted a greater increment in A_net_ than in E. As a result, the relationship between assimilated CO_2_ and transpired water increased linearly, leading plants cultivated under the PCP system with 16,000 stems ha^−1^ to exhibit higher WUE compared with the other densities. On the other hand, WUE in the LS behaved similarly to g_s_ and E, showing no relationship with the increase in stem density ha^−1^.

**Figure 5:**
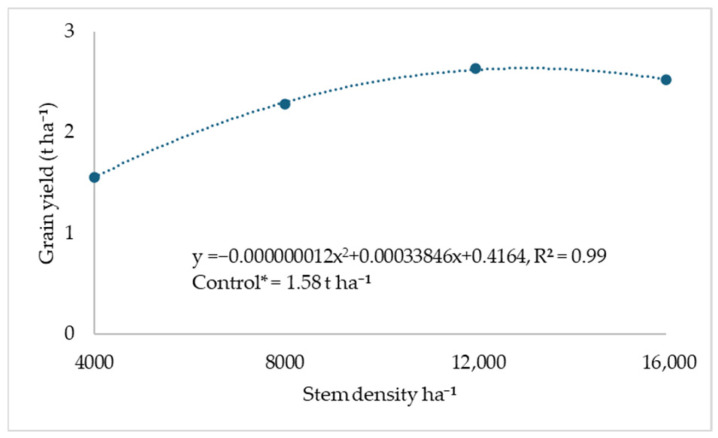
Mean yield over four production cycles (2015 to 2018) in Arabica coffee managed under the PCP system with different stem densities ha^−1^ and under the conventional pruning system (control). (*) Significant (*p* ≤ 0.05). Each value represents the mean ± SE (n = 4).

Grain yield (mean of four harvests) in the PCP system also showed a significant interaction (*p* ≤ 0.05) with stem density ha^−1^ (Figure 5). However, the response was not linear with increasing stem density, as observed for A_net_. Maximum yield, 2.8 t ha^−1^, was achieved at approximately 14,000 stems ha^−1^, representing an increase of 77.5% compared with the yield obtained at 4000 stems ha^−1^ (1.578 t ha^−1^).

## 3. Discussion

Stomatal conductance plays a central role in regulating gas exchange between the plant and the atmosphere [11,12]. In this study, the reduction in gs observed in the upper stratum of control plants was associated with lower Anet and E values, indicating a limitation in gas exchange under the traditional pruning system.

Gas exchange is strongly influenced by stomatal conductance, which responds to environmental conditions and plant physiological status [12,13]. In the present study, reductions in gs were consistently accompanied by decreases in Anet and E in the upper stratum of control plants, whereas under PCP these variables remained more uniform between canopy strata. Even under well-hydrated conditions, stomatal conductance in coffee leaves can be sensitive to increases in air vapor pressure deficit, and moderate VPD values may induce partial stomatal closure depending on plant water status and environmental conditions [14,15,16,17,18,19,20]. However, in the present study, the limitation of leaf gas exchange in the upper stratum of control plants was not clearly explained by VPDleaf/air alone. Therefore, the reduced Anet, gs, and E observed in this stratum likely reflect the combined effects of canopy structure, light distribution, and source–sink imbalance rather than VPD alone. On the other hand, Stomatal conductance is influenced by plant water status and environmental conditions [21,22,23,24,25,26], which in turn depend on the balance between water loss through transpiration and the water supply from the soil to the leaf [25,26]. In this study, the higher transpiration observed in the lower stratum of control plants may be associated with differences in resource distribution within the canopy, contributing to the observed variation in gs, E, and Anet between canopy strata. The variation in leaf gas exchange parameters, found mainly in the control treatment, was associated with differences in plant growth (Figure 3). These differences were associated with r lower net CO_2_ assimilation, stomatal conductance, and transpiration in the upper stratum of control plants.

Lower values for growth variables in the US were already expected, since this stratum contains a large proportion of leaves and branches in early developmental stages. However, the reduced values of leaf gas exchange obtained in the US (control), the poor vegetative development, together with the low leafiness in the middle third of the plants (Figure 2), may have been associated with the influence of the lower stratum of these plants. This indicates that the lower canopy may have exerted a dominant influence on the physiological performance of the upper stratum under the traditional pruning system.

It is believed that the greater leaf production that occurs in the lower portion of plants under the traditional pruning system results in a higher concentration of internal metabolites and photoassimilates in the lower part of the plant and, consequently, a lower concentration in the upper part [2]. This characteristic is known as “girdling”, in which there is intense vegetative development of the “skirt” (lower stratum of the plant) and poor development of the rest of the plant [10]. This condition reflects a source–sink imbalance, in which the lower canopy acts as a dominant sink, which may limit assimilate distribution and growth in the upper portions of the plant.

This pattern is consistent with previous observations in coffee plants, where plagiotropic branches located in the lower canopy act as strong sinks due to their proximity to the root system, concentrating photoassimilates and reducing their availability to upper canopy regions. This imbalance contributes to reduced leaf gas exchange and growth in the upper strata of plants managed under the traditional pruning system.

Such a characteristic was not observed in the PCP system, since pruning allows the correction of problems related to plant architecture and the recovery of plants that do not meet desirable technical and economic standards [5]. The visual evidence of girdling (Figure 2) supports this interpretation, indicating that the concentration of foliage and production in the lower canopy is associated with an uneven distribution of assimilates and reduced lower net CO_2_ assimilation, stomatal conductance, and transpiration in upper canopy regions. The daytime photon irradiance intercepted by leaves can differ by a factor of 25 between the most shaded leaves and sun-exposed leaves in coffee canopies grown under full sunlight [27]. The low red to far-red ratio (R/Fr) alters leaf chlorophyll content [28], since the daytime R/Fr ratio decreases as light passes through the canopy, because photosynthetic pigments absorb red light [29]. In the present study, the higher SPAD values observed in the lower stratum are consistent with these light-related changes within the canopy. This pattern also reflects the structural differences between pruning systems, in which PCP promotes a more uniform canopy architecture, improving light distribution and reducing differences in SPAD readings [30,31,32] and leaf gas exchange between canopy strata.

Chlorophyll content between plant strata was inversely proportional to anthocyanin and flavonoid levels [33], since the activity of antioxidant enzymes is enhanced under conditions of heat stress or intense light [34]. Thus, the synthesis of these compounds was intensified in regions with greater exposure to solar radiation, as observed in the US of the plants. This pattern reflects the greater exposure of the upper canopy to solar radiation, as observed in the present study.

The leaves in the LS (PCP) developed under greater shading conditions caused by the upper stratum of the canopy. In turn, these shade-developed leaves showed higher A_net_ than those developed under normal light conditions [35]. This behavior was repeated with the increase in stem density ha^−1^, in which higher levels of shading favored the photosynthetic process [36]. In this study, the increase in Anet with stem density is consistent with these responses to canopy shading. Moderate increases in stem density may enhance photosynthetic efficiency through partial shading, improving light distribution within the canopy. However, excessive density may lead to self-shading, reducing the availability of radiation to inner canopy leaves and limiting physiological performance. This pattern is consistent with the increase in Anet observed with increasing stem density in the present study.

To increase their carbon fixation potential, shaded plants undergo certain modifications, such as the development of thinner and larger leaves [37], with more thylakoids per granum and more grana per chloroplast [38], higher quantum yield of photosynthesis, and lower light compensation and light saturation points [27]. These modifications allow plants to efficiently capture and use the available light energy, thereby increasing dry matter production [39].

In turn, in the US, g_s_ was higher than in the LS, since light has rapid effects on g_s_, increasing stomatal pore opening. Stomatal conductance is influenced by light availability and environmental conditions [40]. However, even with higher g_s_, the lower VPD_leaf/air_ in the US resulted in a lower transpiration rate than in the LS. Under the measurement conditions used in this study, this response resulted in higher instantaneous leaf-level water-use efficiency, estimated as the ratio between net CO_2_ assimilation and leaf transpiration [41]. This relationship was observed in the present study, where higher WUE was associated with increased stem density under PCP. However, this parameter should be interpreted only as an instantaneous leaf-level gas-exchange indicator, not as whole-plant water-use efficiency.

The increase in stem density ha^−1^ led to an increase in grain yield; however, this response was not linear as observed for A_net_. Maximum yield was obtained at around 14,000 stems ha^−1^, whereas maximum A_net_ occurred at 16,000 stems ha^−1^. This non-linear response of yield to stem density suggests that intermediate densities favor productivity, whereas higher densities may promote competition for light and increased canopy self-shading, resulting in reduced yield. However, such maximum A_net_ is unlikely to be achieved under field conditions with increasing stem density ha^−1^, since leaf shading also increases. Therefore, stem densities above 14,000 stems ha^−1^ did not result in further increases in grain yield. These results indicate that maximum net CO_2_ assimilation does not necessarily correspond to maximum yield under increasing stem density.

In the traditional pruning system, the occurrence of girdling reduces A_net_ in the upper stratum, which may be associated with differences in resource distribution within the canopy. Under PCP, A_net_ increases as stem density per hectare increases. There is lower photo-oxidative stress in the lower stratum compared with the upper stratum, and increasing stem density in PCP enhances leaf shading. No differences in growth parameters were observed between PCP and the traditional system. Overall, the results indicate that changes in canopy architecture induced by PCP are associated with a more balanced distribution of assimilates and greater uniformity of leaf gas exchange within the plant, which may contribute to higher productivity under this management system. However, the higher Anet obtained at 16,000 stems ha^−1^ did not result in greater grain yield, indicating that maximum net CO_2_ assimilation does not necessarily translate into maximum yield under increased stem density. The response of Anet to stem density (Figure 4), although consistent, cannot be fully explained based on the available data and likely reflects combined effects of light distribution, canopy structure, and source–sink interactions.

Finally, our hypothesis was partially supported, as PCP did not significantly increase mean leaf gas exchange rates compared to the traditional system, but it promoted greater spatial uniformity of gas exchange within the canopy and resulted in higher grain yield, indicating that improvements in canopy organization rather than absolute physiological enhancement were the main drivers of productivity gains. However, these findings should be interpreted within the context of the specific environmental and management conditions under which the experiment was conducted. In addition, because the physiological evaluations were performed under controlled conditions and at a single time point, caution is warranted when extrapolating these results to other growing conditions or when inferring plant responses under natural environmental variability.

The results obtained in this study demonstrate that programmed cycle pruning (PCP) promotes improvements in canopy gas-exchange uniformity and yield in Arabica coffee. Although the present study did not include an economic analysis, previous studies conducted under similar experimental conditions have demonstrated that PCP is economically viable and presents lower financial risk compared to traditional pruning systems, mainly due to increased productivity and the absence of zero-yield years [42]. These findings reinforce the potential of PCP as a management strategy capable of improving both agronomic performance and long-term profitability. However, future studies integrating physiological, agronomic, and economic analyses are recommended to provide a more comprehensive evaluation of this system under different production conditions.

## 4. Materials and Methods

### 4.1. Site Description

The study was conducted in the field in the district of Alto Mutum Preto, municipality of Baixo Guandu, in the northwestern region of the state of Espírito Santo, Brazil, at an altitude of 634 m and at the geographic coordinates 19°21′44.32″ S and 40°50′31.95″ W. According to the Köppen climate classification, the area is located in a region with an Aw-type (tropical humid) climate and has an average temperature of 21.4 °C, an average annual rainfall of 1260 mm [43], and an undulating to rugged topography.

### 4.2. Crop Description

The crop was managed under commercial field conditions typical of the region. The experimental field was established with the cultivar Catuaí Vermelho IAC 81, which has late maturation, was 12 years old, spaced at 2.5 m × 1.0 m, and grown under rainfed conditions. Fertilization was carried out according to the recommendations of [44], and crop management practices followed the guidelines of [45]. Each experimental plot consisted of three rows of plants, spaced at 2.5 m × 1.0 m, totaling dimensions of 7.5 m × 9.0 m per plot. The three central plants were considered as the useful area for evaluations in order to reduce border effects. It is important to note that the control treatment represents a traditional management system without recent renewal pruning, whereas PCP treatments correspond to renewed plants. Therefore, comparisons should be interpreted as differences between management systems rather than strictly equivalent structural conditions.

### 4.3. Experimental Design and Statistical Analysis

The experimental design was a randomized block design with four replications. The treatments were arranged in a 4 × 2 factorial scheme with an additional treatment, represented by the combination of four stem densities (4000, 8000, 12,000, and 16,000 stems ha^−1^) and two data collection positions on the plant, corresponding to the lower stratum and the upper stratum of the canopy.

The additional treatment (control) represented the traditional pruning system used in coffee management, in which one stem per plant (sometimes two) is predominant and plagiotropic branches are not removed, with subsequent stump pruning carried out after the plants lose vigor. In this system, plants are allowed to grow freely and are generally stump-pruned after vigor decline (10–15 years). The control treatment represented the traditional pruning system under commercial conditions, in which plants were not subjected to recent renewal pruning and maintained their original canopy structure. Therefore, comparisons between PCP and the control reflect differences between renewed and non-renewed systems under typical field management conditions.

Table 3 presents the combination of treatments with the spacing used in the field, resulting in the final stem density for each treatment.

### 4.4. Implementation of PCP in the Field

**Figure 6:**
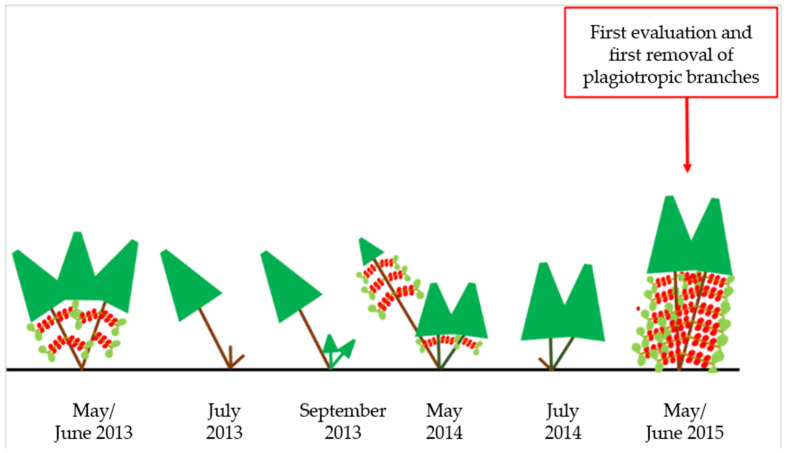
Illustrative scheme of the steps carried out in the experiment for the implementation of programmed cycle pruning in *Coffea arabica* L. ‘Catuaí Vermelho IAC 81’.

In July 2013, after fruit harvest, programmed cycle pruning (PCP) was introduced in the field by removing approximately 75% of the old stems and plagiotropic branches with reduced productive capacity, in order to promote greater light penetration at the plant base and stimulate the emergence of vigorous shoots, as described by Verdin Filho et al. [6]. The first suckering was carried out 50 days after pruning to select the number of shoots corresponding to the number of stems established for each treatment, and the remaining shoots were periodically removed. After fruit harvest in 2014, the stems remaining from the 2013 pruning were eliminated, and the crop was maintained only with the previously selected shoots, resulting in complete renewal of the plant structure. From 2015 onwards, production was established based on these renewed shoots. In 2015, the first harvest of fruits from the stems formed after the implementation of PCP was carried out (Figure 6).

After the initial renewal phase, the PCP system follows a cyclical pattern of stem replacement over successive harvests, maintaining a balance between vegetative growth and production.

### 4.5. Evaluations

#### 4.5.1. Leaf Gas Exchange, SPAD Reading, Anthocyanins, and Flavonoids

The evaluations were carried out between 8:00 a.m. and 10:00 a.m. on sunny days, in order to avoid the effects of high vapor pressure deficit and stomatal closure typically observed during the afternoon. Measurements were performed on the third pair of leaves of the lower plagiotropic branch (third branch from the plant base) and of the upper plagiotropic branch (third branch from the plant apex), alternating between both sides of the planting row.

- Net photosynthetic rate (A_net_), stomatal conductance (g_s_), leaf transpiration (E), and vapor pressure deficit between the leaf and air (VPD_leaf/air_) were determined using an infrared gas analyzer (IRGA (LI-COR Biosciences, Lincoln, NE, USA)), model LI-6400 (LI-COR Biosciences, Lincoln, NE, USA), with an external CO_2_ supply of 400 μL L^−1^, irradiance of 1500 μmol m^−2^ s^−1^, and block temperature of 30 °C. All measurements were performed under the same chamber conditions for every treatment, in order to minimize short-term environmental variability and enable comparison of intrinsic physiological responses among treatments, rather than to reproduce in situ field conditions.
- SPAD reading: Estimated using a portable chlorophyll meter, model SPAD-502 Plus “Soil Plant Analysis Development” (Konica Minolta, Tokyo, Japan). Five readings were taken on each leaf (the same leaves used for gas exchange measurements), and the mean of these five readings was considered the value for each replication.
- Anthocyanin index (ANT-RG) and flavonoids (FLAV): estimated using a Multiplex fluorometer (Force-A, Orsay, France) with multiple light excitation sources (ultraviolet, blue, green, and red). The device was positioned approximately 1 cm from the leaf surface (the same leaves used for gas exchange measurements) for each reading. The physiological data obtained from the Multiplex fluorometer were parameterized prior to analysis, following the manufacturer’s recommendations.

Physiological evaluations were conducted after the stabilization of the canopy structure resulting from the implementation of programmed cycle pruning (PCP). Because PCP is based on gradual canopy renewal over successive production cycles, its effects on plant architecture and source–sink relationships are cumulative rather than immediate. Therefore, measurements performed in 2017 represent a consolidated physiological condition of the system. In addition, the evaluations were carried out in the period preceding the subsequent harvest, allowing the assessment of plant functioning under conditions that directly influence yield formation. This approach ensures that the physiological responses observed are consistent with the established management system and its effects on crop performance.

Three semiannual evaluations were carried out for the variables estimated using the Multiplex (Force-A) and SPAD-502 devices, conducted in December 2015, June 2016, and December 2016. For the variables determined using the IRGA, only one evaluation was performed, in June 2017.

#### 4.5.2. Shoot Biometric Traits and Grain Yield

Biometric analyses and grain yield evaluations were carried out in the 2015, 2016, 2017, and 2018 growing seasons.

In the biometric analyses, canopy diameter, plagiotropic branch length, and number of inflorescences per branch were determined in both the lower and upper strata of the plants.

To obtain the plagiotropic branch length in the lower stratum, four branches were measured immediately after the fourth branch developed from the plant base. The plagiotropic branch length in the upper stratum was obtained by measuring four branches immediately after the fourth branch developed from the stem apex. Branches were measured in each of the four quadrants of the plant, and the final value was expressed as the arithmetic mean of the four measurements. Based on branch length, canopy diameter was also estimated for both the lower and upper strata, considering each branch length as the radius of a circumference; therefore, canopy diameter was equivalent to 2 × branch length. Subsequently, the mean canopy diameter was estimated using the arithmetic mean of the obtained values.

The number of inflorescences per branch in both strata was determined using the same sampling approach described for branch length.

Fruits were harvested based on field assessment of fruit maturation, when the proportion of green fruits was below approximately 20%. All plants within each plot were harvested and individually dried in a suspended drying system. Fruits were manually harvested from all plants within each plot, and the harvested material was subsequently processed and dried under controlled conditions until approximately 12% moisture content. After weighing the grains, yield was expressed on an area basis and converted to t ha^−1^.

### 4.6. Statistical Analysis

The mean of the three semiannual evaluations (for the variables estimated using the Multiplex (Force-A) and SPAD-502) was calculated, and the data were subjected to analysis of variance (ANOVA). The assumptions of normality and homogeneity of variances were verified prior to analysis. The experimental design was a randomized block design, with stem density and canopy stratum considered as fixed effects and blocks as random effects. To compare the factorial and control treatments, data from both canopy strata were considered. Regarding the data collection position on the plant, the means of these treatments were compared using Tukey’s test (*p* < 0.05). For the effects of stem density, the variables were analyzed using regression analysis. The additional (control) treatment was compared through decomposition of the treatment sum of squares into orthogonal contrasts, specifically testing the contrast between the control and the factorial treatments. Statistical analyses were performed using the Genes statistical software (version 2.0).

## 5. Conclusions

The results of this study indicate that the Programmed Cycle Pruning (PCP) system is associated with changes in the physiological dynamics and productive performance of Arabica coffee plants compared with the traditional pruning system. Although vegetative growth variables such as canopy diameter, branch length, and number of inflorescences did not differ between pruning systems, PCP was associated with greater physiological balance within the canopy and reduced the occurrence of the “girdling” phenomenon observed under traditional management. Net photosynthetic rate increased with stem density under PCP, suggesting changes in physiological activity within the plant canopy. In addition, PCP resulted in higher grain yield compared with the traditional system, with maximum productivity occurring at approximately 14,000 stems ha^−1^. These findings suggest that PCP, combined with adequate stem density management, may improve physiological performance and contribute to increased productivity in Arabica coffee cultivation.

## Figures and Tables

**Table 1:** SPAD reading, flavonoid index (FLAV), and anthocyanin index (ANTHR-G) in two plant strata (LS = lower stratum and US = upper stratum) in Arabica coffee under the PCP system and the traditional pruning system (control).

Data Collection Position	SPAD Reading	FLAV	ANTHR-G
LS (PCP)	67.60 (±1.15) a	1.34 (±0.004) b	−0.506 (±0.0009) b
US (PCP)	62.93 (±1.62) b	1.61 (±0.03) a	−0.487 (±0.0022) a
LS (Control)	66.27 (±1.38) ab	1.20 (±0.04) b	−0.511 (±0.0049) b
US (Control)	57.88 (±1.69) c	1.62 (±0.05) a	−0.473 (±0.0081) a
CV (%)	2.54	4.91	1.66

Mean values ± SE (n = 4) followed by the same letter do not differ significantly according to Tukey’s test (*p* ≤ 0.05).

**Table 2:** Pearson correlation between net photosynthetic rate (A_net_), stomatal conductance (g_s_), and transpiration (E) in the upper and lower strata of Arabica coffee managed under the PCP system.

Upper Stratum	A_net_	g_s_	E
A_net_	-	0.9973 **	0.9999 **
g_s_	-	-	0.9973 **
E	-	-	-
**Lower Stratum**			
A_net_	-	0.46 ^ns^	−0.43 ^ns^
g_s_	-	-	0.59 ^ns^
E	-	-	-

** Significant at 1% probability level. ^ns^ not significant.

**Table 3:** Description of the treatments combining stem density and data collection position on the plant, and the additional (control) treatments, for *Coffea arabica* L. ‘Catuaí Vermelho IAC 81’.

Treatments	Data Collection Position	Number of Stems per Plant	Stem Density (Stems ha^−1^)
1	Lower stratum	1	4000
2	Lower stratum	2	8000
3	Lower stratum	3	12,000
4	Lower stratum	4	16,000
5	Upper stratum	1	4000
6	Upper stratum	2	8000
7	Upper stratum	3	12,000
8	Upper stratum	4	16,000
9 (Control 1)	Lower stratum	1 to 2	4000 to 8000
10 (Control 2)	Upper stratum	1 to 2	4000 to 8000

## Data Availability

The original contributions presented in this study are included in the article. Further inquiries can be directed at the corresponding author.
